# Secretory Phospholipase A_2_–Albumin–Membrane Interactions: A Proposed Mechanism of Internal Cell and Tissue Injury in Systemic Inflammation

**DOI:** 10.3390/biom16101461

**Published:** 2026-10-07

**Authors:** Francis H. C. Tsao, Keith C. Meyer

**Affiliations:** Department of Medicine, Division of Pulmonary and Critical Care Medicine, School of Medicine and Public Health, University of Wisconsin, Madison, WI 53792, USA; kcmeyer@wisc.edu

**Keywords:** albumin, cell injury, drug pharmacokinetics, membranes, secretory phospholipase A2, systemic inflammation

## Abstract

Systemic inflammation in sepsis causes internal cell and tissue injury through mechanisms that remain incompletely understood. Secretory phospholipase A2 (sPLA_2_-IIA; hereafter, sPLA_2_), a potent inflammatory mediator whose circulating levels rise dramatically during sepsis, hydrolyzes membrane phospholipids in injured and dying cells, thereby releasing fatty acids and lysophospholipids that further amplify inflammation. Serum albumin—particularly a specific membrane-binding fraction (SFA)—normally acts as a cytoprotective scavenger of these lipid products, thereby maintaining membrane phospholipid homeostasis in concert with sPLA_2_. This perspective synthesizes nearly two decades of work demonstrating that the sPLA_2_–albumin interaction is a critical, underappreciated determinant of internal cell and tissue injury during systemic inflammation. As sPLA_2_ activity rises during sepsis, fatty acids generated from injured membranes progressively saturate albumin’s binding sites, inducing structural misfolding that abolishes its cytoprotective capacity and paradoxically enhances further sPLA_2_-mediated membrane degradation. Serial serum samples from ICU patients with sepsis revealed a statistically significant positive correlation between sPLA_2_ activity and albumin membrane-binding-site saturation, modifiable by nutritional intervention. Age-related albumin misfolding and oxidative stress are identified as important amplifying factors. The sPLA_2_–albumin axis represents a promising target for biomarker development and therapeutic intervention in critically ill patients.

## 1. Introduction

Sepsis is a life-threatening condition caused by a dysregulated host response to infection that leads to widespread systemic inflammation, progressive cell and tissue injury, and ultimately organ failure [1,2]. Despite decades of research and advances in critical care, the global burden of sepsis remains enormous. An estimated 166 million incident cases and 21.4 million deaths were recorded in 2021 alone—nearly one-third of all global deaths that year—and a reversal of prior progress attributable in significant part to the COVID-19 pandemic [3,4]. Mortality rates remain unacceptably high even when infections are identified and treated promptly [5,6]. A central and still incompletely understood feature of sepsis is the extent of internal cell and tissue death that occurs during the systemic inflammatory response—damage that exceeds what can be attributed to the infection itself and that drives the cascade of organ dysfunction responsible for patient mortality [2,7].

Among the mediators implicated in this process, secretory phospholipase A2 (sPLA_2_) occupies a pivotal position. sPLA_2_-IIA (hereafter referred to as sPLA_2_), the predominant circulating isoform during acute inflammation, is released by immune cells, gastrointestinal Paneth cells, and other cell types in response to infection and inflammatory stimuli; circulating levels can increase by as much as 1000-fold in patients with sepsis, surgical trauma, and acute pancreatitis [8,9,10,11]. In the intestinal lumen, sPLA_2_ functions as a defensin-related bactericidal protein; in the systemic circulation, however, it primes neutrophils, promotes the hydrolysis of membrane phospholipids in injured and dying cells, and releases bioactive fatty acids and lysophospholipids that further amplify inflammation [8,12,13,14].

Serum albumin, the most abundant circulating protein, plays an equally important but less appreciated counterpart role. Beyond its well-recognized functions in oncotic pressure and ligand transport, albumin—particularly a specific membrane-binding fraction—acts as a critical scavenger of the fatty acids and lysophospholipids released by sPLA_2_ from injured cell membranes, which helps to maintain membrane phospholipid homeostasis [15,16,17,18]. In sepsis, this protective capacity is progressively lost. As fatty acids are released in quantities that overwhelm albumin’s binding sites, albumin undergoes conformational changes that compromise its structure and function, and this process may enhance further membrane degradation [18,19,20].

The objective of this perspective is to integrate our studies of sPLA_2_, serum albumin, and bilayer membranes into a unified conceptual framework for understanding the regulation of cellular membrane homeostasis during systemic inflammatory conditions. In doing so, we aim to highlight the pathophysiological significance of albumin structural integrity—as distinct from albumin concentration—as a determinant of internal cell and tissue injury in sepsis, and to identify the sPLA_2_–albumin axis as a promising target for future biomarker development and therapeutic investigation in critical illness.

## 2. Secretory Phospholipase A2 as a Mediator of Systemic Inflammation

Phospholipase A2 (PLA2) enzymes comprise a superfamily of lipolytic proteins that catalyze the hydrolysis of the sn-2 ester bond of membrane phospholipids, thereby releasing free fatty acids and lysophospholipids [21,22]. Among the isoforms of PLA2, the calcium-dependent secretory PLA2 enzymes are the most abundant extracellularly and the most relevant to systemic inflammation [8]. sPLA_2_-IIA—a small, cationic, 14 kDa protein—is the predominant isoform detected in human circulation during acute inflammation and is closely correlated with bacterial infection [8,21,22]. Levels are low or undetectable in healthy individuals but can rise by as much as a thousand-fold in septic shock, acute pancreatitis, and surgical trauma [8,11]. Despite three decades of study, including substantial interest as a drug target in chronic inflammatory disease, clinically approved sPLA_2_-targeted therapy has not yet emerged, in part because the relevance of its catalytic versus non-catalytic functions to human disease remains incompletely defined [10].

The ability to measure sPLA_2_ activity sensitively in clinical samples—a task for which conventional titrimetric, colorimetric, and radiometric methods are insufficiently sensitive or impractical—motivated our development of a continuous fluorescence microplate assay using BODIPY-labeled liposomes (50% phosphatidylcholine–50% phosphatidylglycerol) as substrate, a composition that eliminated interfering signals from serum albumin and lipoproteins [11]. Using this assay, we showed that sPLA_2_ activity was markedly elevated in the sera of patients with sepsis compared with healthy subjects [11], and that it sensitively tracked the time course of serum sPLA_2_ activity in response to endotoxin in rats [23], providing the methodological foundation for the studies described throughout this perspective.

The clinical relevance of sPLA_2_-IIA extends beyond bacterial sepsis to other forms of systemic inflammatory injury. In COVID-19, markedly elevated circulating sPLA_2_-IIA levels have been shown to correlate with disease severity and mortality in a pattern paralleling bacterial septic shock. Machine-learning analysis demonstrated that sPLA_2_-IIA levels effectively stratified patients with severe COVID-19 from those who succumbed, outperforming leading cytokines and chemokines as a predictor of ICU outcome [24]. Temporal analysis of serial ICU samples further revealed that sPLA_2_ levels increased progressively over the first 7 ICU days in patients who died but attenuated in survivors [25].

### Compartment-Dependent Roles and Physiological Regulation of sPLA_2_

sPLA_2_’s functional role is profoundly compartment-dependent. In the intestinal lumen, sPLA_2_-IIA is secreted by Paneth cells as a defensin-related antimicrobial protein; its cationic charge enables preferential disruption of the negatively charged membranes of Gram-positive and Gram-negative bacteria as part of innate mucosal immunity [12,26,27,28,29]. In the systemic circulation, however, elevated sPLA_2_ shifts from protective to injurious as it primes neutrophils for augmented inflammatory responses, drives eicosanoid synthesis from liberated arachidonic acid, and hydrolyzes the phospholipids of injured or apoptotic cells in which phosphatidylserine has been externalized to the outer membrane leaflet [8,13,14,30,31].

This duality is further shaped by nutritional and pharmacological variables. In a murine model, luminal sPLA_2_ activity and the bactericidal capacity of intestinal secretions declined progressively as enteral stimulation was reduced, with parenteral nutrition producing the greatest suppression of Paneth cell sPLA_2_ content while simultaneously increasing systemic sPLA_2_ activity when surgical stress was superimposed [26,27]. This dissociation supports the gastrointestinal tract as an important source of circulating sPLA_2_ during inflammatory states and identifies parenteral nutrition as a modifiable risk factor for systemic sPLA_2_ elevation. Independently, prophylactic dietary polyphenolics attenuated endotoxin-induced serum sPLA_2_ elevation in a dose-dependent manner in rats—the only inflammatory marker tested that responded to both challenge and supplementation [23]. Together, these findings establish sPLA_2_ as a dynamically regulated mediator that is protective in the gut lumen but potentially injurious systemically, and the interaction of sPLA_2_ with serum albumin determines the balance between tissue protection and injury during systemic inflammation.

## 3. Serum Albumin: Structure, Function, and Interaction with sPLA_2_

Human serum albumin is the most abundant protein in the circulation, comprising approximately 60–65% of total serum protein at concentrations of 35–50 g/L [15]. Produced exclusively by the liver, albumin is the principal determinant of plasma oncotic pressure, the predominant antioxidant in intra- and extravascular compartments, and the primary transport vehicle for fatty acids, hormones, bilirubin, metal ions, and drugs, via up to seven fatty acid binding sites whose occupancy is accompanied by conformational change in albumin [15,32,33]. Beyond these classical roles, albumin is increasingly recognized as an integrative protein linking the physiology of tissues and organ systems throughout the body, with broad implications for diagnostics, disease pathogenesis, and therapeutics, as well as possessing certain enzymatic activities [16,17,34]. Studies of serum albumin dynamics during sepsis have further shown that albumin concentration during critical illness reflects a complex interplay of acute-phase inflammatory catabolism, capillary leak, fluid redistribution, and hepatic dysfunction, reinforcing the conclusion that serum albumin concentration alone is an inadequate surrogate for its functional state [35].

Albumin’s functional capacity depends not only on its concentration but critically on its structural integrity. In healthy individuals, 70–80% of circulating albumin exists in the reduced, mercaptalbumin form, with the free thiol at Cys-34 conferring antioxidant activity; under oxidative stress, this redox balance shifts toward more oxidized, structurally altered forms [36,37]. Such misfolding is distinct from the quantity-based hypoalbuminemia traditionally monitored clinically and cannot be detected by standard measurements of albumin concentration alone.

Serum albumin has long been recognized to modulate PLA2 activity in in vitro non-fluorescent assay systems employing micelles or liposomes as substrates, primarily by binding lipid substrates or hydrolytic products rather than by interacting directly with the enzyme itself [38]. The nature of these effects was found to be highly assay-system dependent: at low substrate concentrations, albumin produced apparent inhibition of PLA2 through substrate depletion; at higher liposome concentrations, albumin stimulated PLA2 activity by relieving product inhibition; and in cholate-mixed micelle systems, biphasic effects were observed, with stimulation at lower albumin concentrations followed by diminished activity at higher concentrations, attributable to sequestration of fatty acid products from the lipid–water interface [38]. These findings established that protein modulation of PLA2 activity in non-fluorescent assay systems reflects interactions with lipid substrates and products rather than direct enzyme–protein binding [38]. The fluorescent liposome assays developed in our studies extended these earlier observations by enabling sensitive, real-time assessment of albumin’s direct interactions with intact bilayer membranes in the presence of sPLA_2_, thereby revealing cytoprotective albumin–membrane interactions that are structurally dependent and cannot be detected using conventional non-fluorescent approaches [18]. These studies further demonstrated that albumin structural integrity is critical to its pathophysiological role in membrane homeostasis [18,19,20].

### 3.1. The Specific Fraction of Albumin and Its Membrane-Binding Activity

Albumin in serum is not functionally homogeneous. When fractionated by high-performance liquid chromatography, only a small, specific fraction—designated the Specific Fraction of Albumin (SFA)—interacted with membrane phospholipids in a manner facilitated by sPLA_2_, generating a characteristic negative fluorescence signal in our liposome assay that was confirmed by tandem mass spectrometry to be albumin-specific [18]. SFA eluted at a position consistent with mercaptalbumin, suggesting that an intact Cys-34 site is important for membrane-binding activity. Oxidation of albumin with hydrogen peroxide reduced this activity by approximately 50%, while pre-incubation with sPLA_2_-generated fatty acids and lysophospholipids completely abolished it, which indicates that both oxidative modification and fatty acid loading of albumin’s binding sites can eliminate the membrane-protective function of SFA [18]. The fluorescent liposome assay specifically demonstrated that SFA binds to bilayer phospholipid membranes in a manner facilitated by sPLA_2_, revealing a direct, real-time protein–lipid interaction at the membrane surface that reflects SFA’s function as a cytoprotective lipid product scavenger; this structurally dependent interaction could not be detected using the conventional non-fluorescent substrate systems in which albumin’s effects on PLA2 activity are attributable to substrate and product binding [38].

### 3.2. Albumin Misfolding with Aging and in Disease

Using the same liposome assay with substrates of varying membrane fluidity, we found that albumin from the sera of 74-year-old healthy subjects enhanced sPLA_2_ catalytic activity on membrane phospholipids to a markedly greater degree than albumin from 24-year-old subjects, reflecting hydrophobic interactions consistent with exposure of non-polar residues normally buried within native, folded albumin [19,39]. Heat-challenge experiments confirmed that this reflected reduced structural stability with age: the ratio of albumin activity at 80 °C to 22 °C was significantly higher in older subjects than younger subjects (1.634 ± 0.065 vs. 1.035 ± 0.088, *p* < 0.0001) [19]. These findings indicate that albumin in older individuals exists in a partially misfolded and compromised state, likely attributable to cumulative oxidative and other age-related modifications [19,36].

This pattern extended into disease states. In a study spanning young versus older healthy subjects, individuals with mild cognitive impairment, and patients with Alzheimer’s disease (AD), albumin fatty acid binding activity was elevated across all older groups regardless of cognitive status and was 2–3 times higher in cerebrospinal fluid (CSF) than serum from the same individuals, likely reflecting disproportionate misfolding within the CSF compartment [39]. A striking gender-specific pattern also emerged: CSF binding capacity declined progressively from age-matched controls through mild cognitive impairment to AD in women, and was nearly absent in female AD patients, while a similar decline was not observed in males. This finding may help explain the greater prevalence of AD in women [39]. Together, these aging and disease-related observations suggest that the same fundamental process of albumin structural modification operates across a spectrum of conditions, with consequences shaped by tissue compartment characteristics and the accompanying inflammatory or oxidative stimulus.

### 3.3. The Mechanistic Link: How sPLA_2_ and Albumin Interact at the Membrane Interface

Under normal conditions, sPLA_2_ facilitates the interaction of SFA with membrane phospholipids, driving albumin to the membrane surface to bind and remove the fatty acids and lysophospholipids it generates. This provides a cytoprotective partnership that allows orderly membrane repair [18]. However, when albumin’s binding sites become occupied by endogenous fatty acids, oxidative modification, or sPLA_2_-derived lipid products, this partnership is disrupted. Fatty acid loading induces conformational changes that increase albumin’s hydrophobic interaction with membranes, paradoxically enhancing rather than limiting sPLA_2_ catalytic activity [18,19]. This role reversal, from cytoprotective to membrane-destabilizing, depends on albumin’s structural state and binding-site occupancy, which provides the conceptual foundation for the positive sPLA_2_–SFA correlation observed clinically in sepsis.

## 4. The sPLA_2_–Albumin Axis in Sepsis: Clinical Evidence and Analytical Considerations

This clinical relevance was first suggested by observations in septic patients in whom serum sPLA_2_ activity was markedly elevated while SFA activity (normally negative, reflecting functional membrane binding) shifted to positive values, indicating loss of binding capacity and emergence of a hydrophobic, misfolded albumin phenotype [18].

### 4.1. The sPLA_2_–SFA Correlation in Serial Samples from ICU Patients

To test this relationship, we analyzed 88 sequential serum samples from 20 patients with defined sepsis enrolled in a controlled nutritional feeding trial and performed blinded assays for sPLA_2_ activity, SFA activity, and albumin fatty acid binding activity [20]. sPLA_2_ and SFA activity showed a significant positive correlation across all samples (Pearson r = +0.48, *p* < 0.0001, n = 88) [20]. In our fluorescent assay, negative SFA values indicate intact membrane-binding capacity of native albumin, whereas values shifting from negative toward zero and then positive reflect progressive loss of this cytoprotective function; a positive Pearson r between sPLA_2_ and SFA values therefore reflects an inverse functional relationship in which rising sPLA_2_ activity was associated with a shift in SFA from negative (functional) toward positive (dysfunctional) values, indicating progressive loss of membrane-binding capacity [20]. This relationship was specific to sepsis: it was not observed in healthy subjects or in patients with idiopathic pulmonary fibrosis without active infection, in whom sPLA_2_ remained near baseline and SFA values stayed negative [20].

Serial serum samples were collected from each patient at defined time points anchored to the date of ICU randomization, yielding a mean of 4.4 samples per patient (range 2–7) over the ICU course, as detailed in reference [20]. We note, however, that the 88 samples were serially collected from 20 patients and are therefore not statistically independent at the level of individual observations; the pooled Pearson r should be interpreted as exploratory and hypothesis-generating rather than confirmatory. A mixed-effects model with a random intercept per patient, reporting within-patient and between-patient variance components separately, represents the statistically appropriate approach for definitive analysis and is identified as a priority for re-analysis of the existing dataset [20]. In addition, a cross-lagged analysis testing whether sPLA_2_ rises temporally precede declines in SFA functional capacity—as our mechanistic model predicts—would provide more direct support for the proposed causal sequence and should be incorporated into the design of future prospective studies with defined serial sampling schedules.

### 4.2. Pre-Bound Fatty Acids as the Mechanism of Impairment

To test directly whether albumin’s binding sites were occupied by pre-bound fatty acids, we used spermidine, a polyamine that competes for the electrostatic interactions required for fatty acid binding, as a probe of site occupancy [20]. Spermidine inhibited fatty acid binding by ~50% in fatty acid-free albumin and 30–40% in healthy serum but had no inhibitory effect in septic patients with positive SFA values (indicating complete site saturation), and only ~10% inhibitory effect in septic patients with less severely affected, still-negative SFA values [20]. These results provide direct functional evidence that albumin’s fatty acid binding sites become progressively saturated by sPLA_2_-derived fatty acids during sepsis, driving the misfolding that enhances rather than inhibits further sPLA_2_-mediated membrane degradation [18,20].

### 4.3. Nutritional Modulation of the sPLA_2_–Albumin Axis

The samples above were drawn from a randomized trial comparing permissive underfeeding with standard enteral feeding in critically ill adults with sepsis, which found no difference in mortality or ICU length of stay between the two feeding strategies [40]. Our molecular analysis, however, revealed a difference that conventional outcome measures had missed. In the permissive underfeeding group, sPLA_2_ activity declined more rapidly and to a significant degree over time as compared to the standard feeding group (*p* = 0.0092), while SFA activity showed a corresponding trend toward recovery not seen in the standard feeding group (*p* = 0.046) [20]. This suggests that permissive underfeeding was associated with more rapid resolution of systemic inflammation that was detectable through this molecular axis even without differences in clinical endpoints, and this finding is consistent with our earlier evidence that nutritional variables modulate sPLA_2_ activity [23,26].

### 4.4. sPLA_2_, Albumin, and the Broader Landscape of Sepsis Biomarkers

Currently used sepsis biomarkers (procalcitonin, C-reactive protein, sTREM-1, and white blood cell count) are valuable but largely reflect infection severity at a single time point rather than the downstream consequences for cell and tissue integrity [41,42]. Indeed, sPLA_2_-IIA itself has been proposed as a candidate diagnostic biomarker for sepsis and bacterial infection in a systematic review of the literature [43], underscoring growing interest in this enzyme beyond its role as an inflammatory mediator. The sPLA_2_–SFA dual assay extends this further by capturing the dynamic interplay between an inflammatory enzyme driving membrane injury and a transport protein whose capacity to contain that injury is progressively overwhelmed. This dimension of sepsis pathophysiology is not captured by commonly used biomarkers. The dual assay, like the candidate biomarker role of sPLA_2_ itself, requires validation in larger and more diverse patient cohorts before clinical translation can be considered [43].

Beyond bacterial infection, mounting evidence supports a role for sPLA_2_-IIA in the pathobiology of COVID-19, in which circulating levels parallel those seen in bacterial septic shock, correlate with indices of organ dysfunction and mortality, and outperform cytokines and chemokines as predictors of ICU outcome [24,25]. This raises the possibility that the sPLA_2_–albumin axis identified here may contribute to cell and tissue injury across a broader spectrum of systemic inflammatory states, including pandemic viral illness, thereby expanding its potential significance as both a biomarker and a therapeutic target.

## 5. A Proposed Mechanism: sPLA_2_–Albumin–Membrane Interactions as a Determinant of Internal Cell and Tissue Injury

Taken individually, the evidence above establishes that (1) sPLA_2_ activity is markedly elevated and dynamically regulated during systemic inflammation; that (2) albumin possesses a specific membrane-binding fraction whose cytoprotective activity is abolished in sepsis; that (3) albumin misfolds with advancing age and disease; and that (4) sPLA_2_ and albumin exhibit a consistent relationship in septic patients, with pre-bound fatty acids as the proximate mechanism of impaired albumin function. Taken together, these findings support a unified model in which the sPLA_2_–albumin interaction is a central, previously underappreciated determinant of internal cell and tissue injury during systemic inflammation, as illustrated schematically in Figure 1.

### 5.1. The Normal Partnership and Its Disruption

Under normal physiological conditions, low circulating sPLA_2_ participates in the orderly clearance of membrane phospholipid degradation products from apoptotic cells, thereby facilitating SFA’s interaction with the membrane surface to bind and remove the fatty acids and lysophospholipids generated [11,14,18,30]. Two conditions must exist for this cytoprotective partnership to adequately function: (1) enough binding sites on albumin must be unoccupied, and (2) albumin must be in its native molecular conformation. These conditions both exist under normal physiological conditions [18,31,32].

However, if systemic inflammation ensues, both conditions are progressively violated. Circulating sPLA_2_ levels can quantitatively rise a thousand-fold in septic shock, hydrolyzing not only apoptotic membranes but those of cells injured by the inflammatory process, thereby releasing fatty acids and lysophospholipids that rapidly overwhelm albumin’s binding capacity [8,11,30,42]. Structurally, as binding sites become occupied by these pre-bound fatty acids, albumin undergoes conformational change consistent with misfolding; rather than coating membranes protectively, misfolded albumin enhances membrane accessibility to sPLA_2_ hydrolysis, which amplifies the process that intact albumin would normally contain. This generates the functional signature that underlies the positive SFA values observed in septic patients [11,18,19]. The result is a self-amplifying cycle: elevated sPLA_2_ generates fatty acids; fatty acids saturate and misfold albumin; misfolded albumin enhances further sPLA_2_ activity; and further membrane injury releases more fatty acids. This cycle may operate largely below the threshold of clinical detection while the host’s capacity for membrane repair is progressively exhausted until the burden of injury exceeds any remaining cytoprotective capacity and overt organ dysfunction ensues.

### 5.2. Modifying Factors: Age, Nutrition, and Oxidative Stress

Three factors of clinical relevance are predicted to influence the pace and severity of this cycle. First, advancing age is associated with progressive albumin misfolding independent of acute inflammation [19,39], potentially leaving older patients with reduced functional reserve when sepsis develops, which may represent a plausible contributor to their greater vulnerability to sepsis-related morbidity and mortality. Second, nutritional route and quantity modulate sPLA_2_ activity in both the gut lumen and systemic circulation: parenteral nutrition suppresses protective luminal sPLA_2_ while increasing vulnerability to systemic elevation of sPLA_2_ levels [11,23], while permissive underfeeding and dietary polyphenolics were each associated with attenuated systemic sPLA_2_ activity [20,23]. Third, oxidative stress (from inflammation, aging, or underlying disease) directly impairs albumin’s structural integrity via modification of Cys-34 and other residues [18,36,37,44], such that patients with pre-existing oxidative burden, including the elderly and those with chronic inflammatory or neurodegenerative disease, may enter a systemic inflammatory state with albumin molecules that are already structurally compromised, compounding the effect of acutely elevated sPLA_2_ [19,39].

A related nuance concerns compartmentalized responses: the same suppression of protective luminal sPLA_2_ that accompanies parenteral nutrition and surgical stress coincides with elevation of injurious systemic sPLA_2_ [26,27], simultaneously increasing vulnerability to bacterial translocation from the gut and to systemic membrane injury from the very enzyme that, in its proper compartment, would have contained the bacterial threat.

### 5.3. What the Model Explains, and What Remains to Be Established

This model offers a molecular explanation for why albumin’s functional capacity declines in sepsis independently of nutritional status and hepatic synthesis. Functional capacity declines because albumin is being consumed as it scavenges sPLA_2_-generated lipid products. Measurable signatures indicative of this process consist of the positive sPLA_2_–SFA correlation and pre-bound fatty acid occupancy, and these signatures can track this process over a patient’s clinical course. At the same time, a direct causal demonstration that interrupting the cycle improves outcomes has not yet been achieved, and the patient cohorts studied were relatively small. The relative contribution of this mechanism versus other pathways of cell death in sepsis remains to be characterized. These are tractable questions for future work, for which the assay tools developed here provide a practical platform.

The fluorescent assays described above may have translational utility in the context of intravenous albumin replacement therapy—a common intervention in the management of critically ill patients with sepsis and hypoalbuminemia—by enabling real-time monitoring of the structural and functional integrity of administered albumin preparations, including their membrane-binding capacity and the degree of fatty acid binding-site occupancy. Because the cytoprotective benefits of albumin infusion may depend partly on the membrane-binding capacity of the administered albumin rather than simply on restoration of plasma protein concentration, functional assessment of administered albumin could provide clinically meaningful information regarding the therapeutic value of albumin replacement in individual patients. Emerging narrative reviews of albumin use in critically ill patients further underscore that albumin’s non-oncotic functions—encompassing drug binding, antioxidant activity, and endothelial stabilization—are central determinants of its clinical value, and that functional assessment of administered albumin preparations may be essential to realizing their full therapeutic potential [35].

### 5.4. Albumin Drug Binding, Pharmacokinetics, and Implications for Therapeutic Strategy

The progressive saturation of albumin’s fatty acid binding sites by sPLA_2_-generated lipid products has direct consequences for drug pharmacokinetics in critically ill patients that extend beyond albumin’s role as a cytoprotective scavenger. Human serum albumin is the principal carrier of the majority of drugs administered in the ICU, with two major high-affinity drug-binding sites—Sudlow site I (subdomain IIA, accommodating warfarin, bilirubin, and large anionic compounds) and Sudlow site II (subdomain IIIA, accommodating ibuprofen, diazepam, and aromatic carboxylic acids)—and seven distributed fatty acid binding sites that overlap partially with both drug-binding regions [32,33]. When sPLA_2_-derived fatty acids progressively occupy these sites during sepsis, the resulting conformational changes alter the geometry and electrostatic character of the Sudlow sites, reducing drug-binding affinity and increasing the free (unbound) fraction of co-administered drugs [35]. Since only the unbound drug fraction is pharmacologically active and subject to renal clearance, fatty acid saturation of albumin can simultaneously increase bioavailable free drug concentrations while accelerating elimination—a combination that may produce dose-dependent toxicity at peak and subtherapeutic exposure at trough for highly protein-bound agents such as certain antibiotics, anticoagulants, and antifungals [35]. The principal binding sites of human serum albumin, their key physiological and pharmacological ligands, and the effects of sPLA_2_-driven fatty acid saturation and structural misfolding on each site are summarized in Table 1.

This pharmacokinetic perturbation is distinct from hypoalbuminemia per se, which is separately recognized as a major determinant of drug pharmacokinetics in critically ill patients [42]. The framework proposed here identifies an additional, qualitative dimension of albumin dysfunction—structural misfolding driven by sPLA_2_ activity—that impairs drug binding independently of albumin concentration and that cannot be captured by standard serum albumin measurement alone. The functional assays described in this perspective (SFA membrane-binding capacity and albumin fatty acid binding-site occupancy) could therefore provide clinically actionable information about drug-binding capacity in individual patients, complementing therapeutic drug monitoring programs already standard in critical care.

From a drug discovery and therapeutic strategy perspective, the sPLA_2_–albumin axis offers several tractable intervention points. First, sPLA_2_-IIA inhibitors—including varespladib (LY315920) and related compounds—have been evaluated in clinical trials for inflammatory diseases and have attracted renewed interest following evidence that markedly elevated sPLA_2_-IIA levels correlate with organ dysfunction and mortality in both bacterial sepsis and COVID-19 [24,25]. Although non-selective sPLA_2_ inhibition has not proven beneficial in broad sepsis trials, the mechanistic model developed here suggests that patient selection guided by sPLA_2_ activity and SFA functional status could identify a subgroup—those in the early, escalating phase of the pathological cycle before irreversible albumin misfolding is complete—most likely to benefit from sPLA_2_-targeted intervention. Second, the recognition that albumin’s cytoprotective capacity depends on its structural integrity as well as its concentration raises the therapeutic possibility that albumin preparations enriched in the functionally intact mercaptalbumin fraction (with reduced, unmodified Cys-34) might confer superior efficacy in sepsis compared to standard commercial albumin preparations, whose proportion of oxidized and dysfunctional forms increases with storage. Third, antioxidant strategies targeting Cys-34 preservation—including N-acetylcysteine, reduced glutathione, and related thiol-replenishing agents—represent a pharmacological approach to protecting albumin’s structural and functional integrity during systemic inflammation, an approach mechanistically supported by the central role of Cys-34 oxidation in initiating misfolding [18,19,36,44] and by broader evidence linking protein oxidative damage to progressive neurodegeneration [44]. Collectively, these considerations position the sPLA_2_–albumin axis as a multifaceted therapeutic target whose full clinical exploitation will require functional—not merely quantitative—assessment of albumin in critical illness.

## 6. Conclusions and Future Directions

The studies summarized in this perspective, spanning nearly two decades and a series of progressively refined fluorescent assays, support a mechanistic model in which interactions among sPLA_2_, serum albumin, and cellular membranes play a central, underappreciated role in internal cell and tissue injury during systemic inflammation. The development of a sensitive serum sPLA_2_ assay, studies of its nutritional and pharmacological regulation, the discovery of albumin’s specific membrane-binding fraction and its impairment in sepsis, the characterization of age- and disease-related albumin misfolding, and an exploratory positive sPLA_2_–SFA correlation in serial ICU samples all support a mechanism in which albumin structural integrity regulates its membrane interactions and modifies sPLA_2_ activity during systemic inflammation.

The central insight is that albumin’s role in systemic inflammation extends well beyond passive transport or a marker of nutritional and hepatic functional status. Its structurally intact, membrane-binding fraction is an active participant in containing sPLA_2_-mediated membrane injury, and the progressive loss of this capacity—driven by fatty acid saturation and consequent misfolding—may represent a critical tipping point between controlled inflammation and uncontrolled tissue damage and organ failure. Simultaneous measurement of sPLA_2_ activity and albumin membrane-binding capacity offers a dynamic window into this process that conventional biomarkers do not provide. The recognition that sPLA_2_-generated fatty acid saturation also impairs albumin’s drug-binding capacity, with implications for pharmacokinetics, therapeutic monitoring, and drug discovery as detailed in Section 5.4 and summarized in Table 1, further extends the translational significance of this axis to the broader management of critically ill patients.

Several future directions follow naturally. Clinical validation of the sPLA_2_–SFA relationship in larger, more diverse cohorts—across sepsis etiologies, severity levels, and comorbidities—is the most immediate priority needed to establish generalizability and define clinically predictive thresholds. Mechanistic studies testing whether restoring albumin binding capacity—through nutritional strategies, plasma exchange, or pharmacological sPLA_2_ inhibition—can interrupt the cycle and reduce tissue injury would address the central unanswered causal question. The association between permissive underfeeding and more rapid normalization of both sPLA_2_ and SFA activity also warrants prospective testing in nutritional trials designed specifically around these molecular endpoints.

The fluorescent assays developed over the course of this work—for sPLA_2_ activity, SFA membrane-binding capacity, and albumin fatty acid binding activity—are practical, inexpensive, and adaptable to high-throughput clinical laboratories. Their application to larger populations and integration with existing sepsis biomarker panels represents a tractable, potentially high-yield avenue for advancing both the understanding of systemic inflammation and the precision with which critically ill patients are monitored and managed.

Ultimately, the significance of the proposed sPLA_2_–albumin axis lies not only in what it reveals about the molecular events driving internal cell and tissue injury in sepsis, but in the therapeutic implications that follow from a clearer mechanistic understanding. If the progressive incapacitation of albumin’s cytoprotective function by sPLA_2_-generated fatty acids is indeed a critical driver of tissue damage in systemic inflammation, then interventions that preserve or restore that function—through nutritional strategies, antioxidant supplementation, functionally assessed albumin replacement, or targeted sPLA_2_ inhibition—may offer new approaches to limiting organ injury and improving survival in one of the most challenging and costly conditions in critical care medicine. The parallel development of clinically validated fluorescent assays for sPLA_2_ activity and albumin functional integrity would represent an important step toward translating these mechanistic insights from the research laboratory into bedside practice.

## Figures and Tables

**Figure 1:**
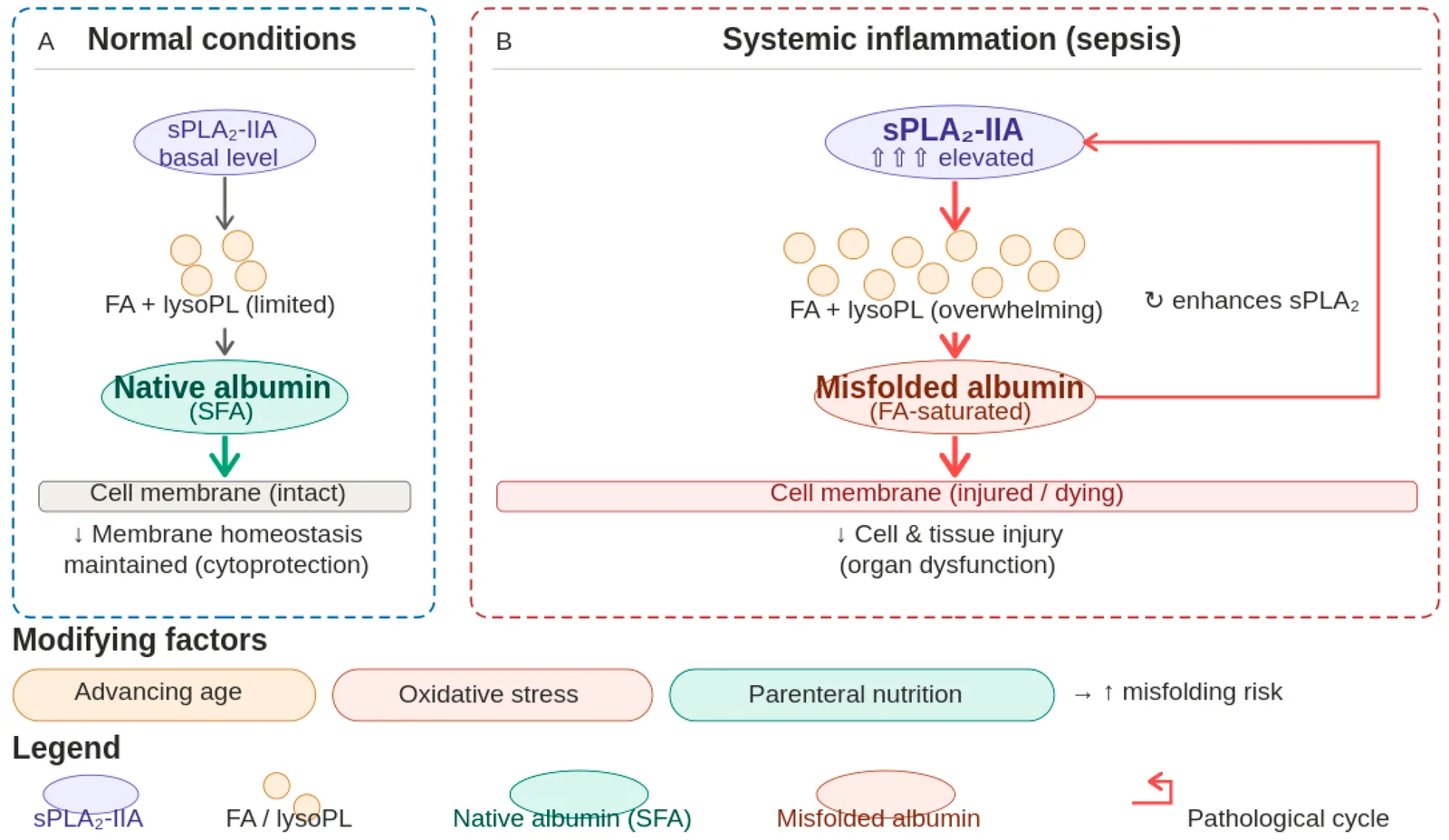
Proposed model of sPLA_2_-IIA–albumin–membrane interactions under normal conditions and during systemic inflammation. Fatty acids (FA) and lysophospholipids (lysoPL) are shown as amber circles; the Specific Fraction of Albumin (SFA) and the cell membrane are color-coded as indicated in the legend. Panel (**A**) (Normal conditions). Basal circulating sPLA_2_-IIA generates a limited quantity of FA and lysoPL through hydrolysis of membrane phospholipids. Native SFA binds these products via unoccupied binding sites, maintaining membrane phospholipid homeostasis. The green arrow indicates the cytoprotective pathway; the teal outcome box indicates maintenance of membrane homeostasis (cytoprotection). Panel (**B**) (Systemic inflammation/sepsis). Circulating sPLA_2_-IIA rises dramatically (↑↑↑, up to 1000-fold in septic shock). The resulting massive release of FA and lysoPL overwhelms albumin’s binding capacity. Albumin misfolds as its binding sites become FA-saturated, abolishing its cytoprotective membrane-binding function. The red arrow on the right side of Panel (**B**) illustrates the self-amplifying cycle: misfolded albumin enhances rather than suppresses further sPLA_2_ activity, driving additional membrane hydrolysis, greater FA and lysoPL release, and further albumin misfolding. The outcome (cell and tissue injury, organ dysfunction) is indicated in red. Lower panel—Modifying factors. Advancing age, oxidative stress, and parenteral nutrition each independently increase albumin misfolding risk and/or systemic sPLA_2_ elevation, thereby amplifying the pathological cycle. See legend panel for color coding of all elements.

**Table 1:** Principal human serum albumin binding sites: key ligands, effects of sPLA_2_-driven fatty acid saturation and misfolding, and clinical relevance in systemic inflammation.

Binding Site	Key Ligands	Effect of FA Saturation/Misfolding	Clinical Relevance in Systemic Inflammation
Sudlow site I (subdomain IIA; drug site 1)	Warfarin, bilirubin, aspirin, phenylbutazone, large anionic NSAIDs	Conformational change reduces binding affinity; displacement of anionic drug ligands by excess fatty acids	Increased free-drug fraction of anticoagulants and anti-inflammatory agents; altered dosing requirements in sepsis
Sudlow site II (subdomain IIIA; drug site 2; overlaps FA sites 3–4)	Ibuprofen, diazepam, thyroxine, medium-chain fatty acids	FA sites 3–4 occupied by sPLA_2_-released fatty acids, competing with and displacing drug ligands	Unpredictable benzodiazepine and NSAID binding; altered sedation and analgesia thresholds in ICU patients
Fatty acid binding sites 1–7 (distributed across domains I–III)	Long-chain fatty acids; hydrophobic drugs (partially)	Progressive saturation by sPLA_2_-released FA drives structural misfolding and Sudlow site distortion	Core mechanism of the sPLA_2_–albumin pathological cycle; SFA assay-measurable marker of binding-site occupancy
Cys-34 free thiol (subdomain IA)	Reactive oxygen species, NO, H_2_O_2_, electrophilic compounds	Oxidation to sulfenic/sulfinic acid eliminates antioxidant scavenging; accelerates structural misfolding	Loss of circulating antioxidant capacity in sepsis and aging; Cys-34 redox status as functional biomarker
N-terminal metal-binding site (subdomain IA)	Cu^2+^, Ni^2+^, Zn^2+^	Metal-binding capacity impaired by conformational change accompanying misfolding	Disturbed trace-metal homeostasis; potential contribution to oxidative stress amplification in sepsis

## Data Availability

No new data were generated or analyzed in this perspective article.
